# Asymmetries in Distractibility: Left Distractors Improve Reaction Time Performance

**DOI:** 10.1038/s41598-018-23498-w

**Published:** 2018-03-26

**Authors:** Nicole A. Thomas, Michael E. R. Nicholls

**Affiliations:** 1 College of Healthcare Sciences, James Cook University, Cairns, Australia; 2 College of Education, Psychology, and Social Work, Flinders University, Adelaide, Australia

## Abstract

Research using the irrelevant-distractor paradigm shows perceptual load influences distractibility, such that distractors are more likely to be processed and decrease reaction times during low perceptual load. In contrast, under high load, attentional resources are limited, and the likelihood of distractibility is decreased. We manipulated distractor placement to determine whether location differentially influenced distractibility. During low load, reaction times were increased equally for all distractor locations. Under high load, left distractors speeded reaction times significantly more than right distractors. We suggest two potential explanations: (1) the central focus of attention was sufficiently large to encapsulate both the distractor and the visual array during low perceptual load, leading to increased distraction—during high load, attention was split across the two visual stimuli, allowing the distractors and array to be processed independently; (2) superior executive control for stimuli in the left visual field allowed participants to ‘catch and release’ left distractors more efficiently, ultimately decreasing distraction and providing a performance benefit. Our findings represent an intriguing development in relation to visual asymmetries in distractibility.

## Introduction

At any given moment, we are faced with innumerable amounts of information, coming from a variety of sources, across modalities. Importantly, humans rely heavily on visual information and must therefore be able to deduce, with high accuracy, which information is relevant and which information should be ignored. From the initial stages of visuospatial processing, focused attention is required for attentional selection and to process information^1,2^. The importance of focused attention is highlighted by the dangers distraction creates. Individuals who experience more attentional failures are indeed at an increased risk for accidents^3–7^.

The irrelevant-distractor paradigm^2,8–11^ provides a robust and reliable measure of distractibility across a wide range of attentional tasks. As the name suggests, this paradigm examines how entirely irrelevant distractors capture attention—and furthermore, failures to orient attention can be measured using this method. During the task, participants are asked to perform a visual search task for one of two given letters, and performance decrements in the presence of cartoon character distractor figures are assessed. The cartoon characters are, of course, entirely irrelevant to the visual search task; their visual appearance, meaning and location are peripheral to the task at hand. As such, the cartoon characters draw attentional resources away from the primary task, creating a potential distraction for participants.

Recently, Forster and Lavie^2^ used this paradigm to establish a measure of the attention-distractibility trait and determine whether attentional focus changes in conditions of high perceptual load. Prior research has shown that conditions of high load significantly reduce distraction because attentional resources are devoted to the primary task, leaving fewer extra resources to process irrelevant stimuli^11–14^. Overall, they found that performance was slower in the presence of distractors. Importantly, the level of distractibility differed between the perceptual load conditions. During low load, irrelevant distractors caused a significant slowing in reaction times, whereas similar performance decrements were not observed in the high perceptual load conditions. This finding is consistent with prior research showing that distractor effects are reduced during high perceptual load, as there are fewer attentional resources available to process distractors^11–14^. Forster and Lavie^2^ presented distractor figures either above or below the visual search array; however, they did not examine whether the location of the distractor influenced distractibility or task performance.

We were interested to further examine the influence of irrelevant distractors by manipulating the location of the distractor figure. Indeed, there are well known differences in the horizontal distribution of attention, such that attention is directed slightly to the left of centre^15^. This asymmetry occurs as a result of bilateral representation of visuospatial attention within the right hemisphere, whereas the left hemisphere represents space contralaterally^16–18^. Namely, each hemisphere generates a spatial bias toward the contralateral visual field, and these biases are balanced through reciprocal inhibition. As a result of interhemispheric competition, the spatial bias of one hemisphere may become dominant and create a stronger attentional weighting toward the contralateral visual field. These recent models of attention highlight the relative contributions of each hemisphere and provide strong evidence that although both hemispheres contribute to visuospatial attention, in most instances the right hemisphere plays a stronger role^16–18^.

In contrast, visual search performance is superior in the right visual field^19–23^. The right hemisphere is superior at global and coordinate processing and also shows a bias for low temporal frequency information, whereas the left hemisphere is advantaged in processing local and categorical information, with a preference for high temporal frequency information^24^. These hemispheric processing differences result in performance differences within the left and right visual fields. Given there are attentional and perceptual differences based on visual field positioning, it is possible that distractors differentially draw upon attentional resources, based upon their location. In particular, attentional biases toward the left side of space might lead left visual field distractors to be more salient, and therefore more distracting.

Similarly, upper and lower visual field stimuli show differences in their ability to attract attentional resources^24–31^, and furthermore, differentially increase distraction^32–34^. In general, stimuli in the upper visual field appear to attract more attentional resources, as a result of their higher salience^35^. In keeping with the horizontal visual field differences reported above, high spatial frequency information, visual search, and local/categorical processing are superior in the upper visual field^24,31^. In contrast, low spatial frequency information, global motion, and global/coordinate processing are superior in the lower visual field^24,31^. As stimuli in the upper visual field receive more selective attention, and visual search performance is superior in the upper visual field, distractors in the upper visual field might be more distracting. Indeed, prior research examining distractibility in attentional asymmetries has shown that upper visual field distractors preferentially capture attention and also draw attention toward the left side of space^32–34^.

Using the method of Forster and Lavie^2^, we modified the location of the distractors so that they appeared either: above, below, to the left, or to the right of the visual search array. In contrast to this, Forster and Lavie presented distractor figures above and below centre only. We expected a main effect of distractor, such that reaction times would be slower in the presence of distractors as compared to when they were absent, replicating Forster and Lavie^2^. In addition, we expected distractor location to differentially impact performance based on perceptual load condition.

In the low load condition, we believed that left visual field distractors would capture attention more than right visual field distractors. Therefore, left distractors were expected to hinder performance in the low load condition. In contrast, we expected that upper visual field, as compared to lower visual field, distractors in the low load condition would attract more attention and therefore be more detrimental to performance.

For the high perceptual load condition, we anticipated that attention would be shifted toward the left, as leftward attentional biases are stronger during more difficult tasks^15–18,33,36,37^. Due to reduced distractibility in the presence of distractors during high load^2^, reaction time should be faster for left distractors as compared to right ones. Similarly, we believed that upper space distractors would be more attention grabbing and would lead to a greater reduction in distractibility as compared to lower visual field distractors.

## Method

### Participants

Thirty-six undergraduate Flinders University students (19 males) completed the experiment in exchange for course credit. Using G-Power, we determined the necessary sample size for an effect size of 0.25 (small), with an alpha level of 0.05 and a power level of 0.95. This calculation dictated we needed 36 participants. All participants had normal or corrected-to-normal vision. According to the FLANDERS handedness survey^38^, seven participants were left-handed (overall *M* = 5.50, *SD* = 7.43). The Flinders University Social and Behavioural Research Ethics Committee granted ethical approval and the experiment was performed in accordance with the ethical standards of the 1964 Declaration of Helsinki.

### Apparatus

Stimuli were presented on a 17′′ LCD, at a distance of 500 mm, using E-prime 2.0 software (Psychology Software Tools, Inc.; www.pstnet.com/E-prime/e-prime.htm). An adjustable chin-rest controlled the viewing angle and ensured the eyes were in line with the middle of the screen. Participant responses were recorded using a model 200 A PST Serial Response Box, placed in line with the midsagittal plane. Participants were monitored via a closed-circuit video system to ensure they remained in the chin rest and completed the task without interruption (e.g., on their mobile phone).

### Stimuli

**Figure 1:**
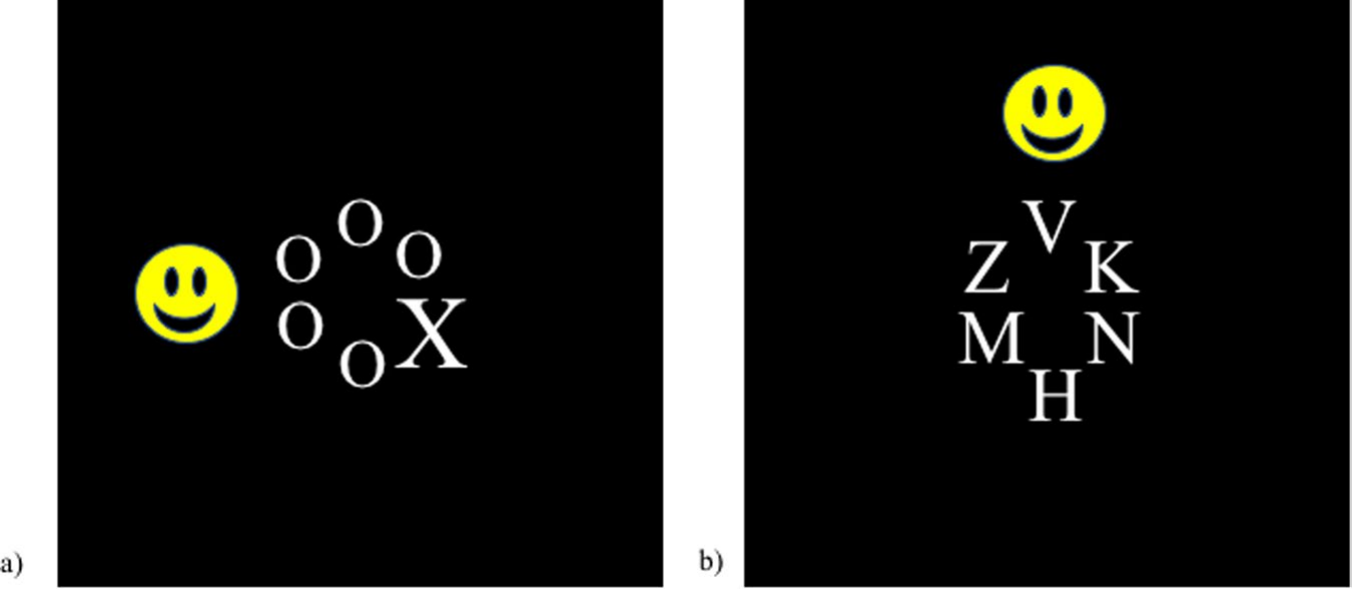
Example stimulus presentation: (**a**) depicts the low perceptual load condition, shows an “X” target, and has a left visual field distractor; (**b**) depicts the high perceptual load condition, shows an “N” target, and has an upper visual field distractor. A generic cartoon face has been used in our figure to avoid copyright violations in publishing our true cartoon stimuli.

Stimuli were obtained from Forster and Lavie^2^ and therefore the visual search array and cartoon distractors were identical to those used in their experiment (see Fig. 1). The visual search array consisted of six letters in a circular formation (radius = 1.6° visual angle). On each trial, one letter was a target: an ‘X’ or an ‘N’ (0.6° × 0.4°), making it a choice reaction time task. Participants were instructed to respond as quickly as possible, pressing the left key if the target was an ‘X’ or pressing the right key if the target was an ‘N’. The non-target stimuli differed across the two load conditions, making the low load condition a feature search and the high load condition a conjunction search. In the low perceptual load condition, all non-target stimuli were lower case ‘o’ letters (0.15° × 0.12°), whereas in the high perceptual load condition, the non-targets were heterogeneous angular letters (randomly selected from ‘K’, ‘V’, ‘W’, ‘Z’, ‘M’, ‘H’) with the same dimensions as the target letter. All letter stimuli appeared in gray on a black background.

The visual search task appeared alone (i.e., distractor absent trials) on 80 percent of trials. The frequency of distractor trials (20%) differed slightly from Forster and Lavie (who used a 75/25 ratio) as we presented task irrelevant distractors in 4 possible locations. Distractors were presented at 4.6° visual angle from the centre of the screen, leaving a distance of 0.6° between the distractor and the array. Distractor stimuli were one of six possible cartoon characters: Donald Duck, Mickey Mouse, Pikachu, Spiderman, SpongeBob SquarePants, and Superman (height between 2.8° and 4°; width between 2.3° and 3.2°).

All participants viewed 3 example trials, of both the high and low perceptual load conditions, where the presentation time of the array was slowed to 10,000 ms to ensure clarity of instruction. Following this, participants completed 12 practice trials in each load condition, which employed the same trial durations as the experimental trials. Participants then completed 4 blocks of 60 trials in each load condition (*n* = 240 trials per load condition). Within each load condition, 192 trials were distractor absent, and 48 were distractor present (*n* = 12 per distractor location). We note that the inclusion of 4 distractor locations inevitably increased the number of trials our participants completed, as compared to Forster and Lavie^2^. Furthermore, our experiment took approximately 60 minutes to complete and, therefore it was not possible to increase trial numbers further and maintain relative consistency with Forster and Lavie’s methodology.

In distractor present trials, each distractor figure was presented twice in each distractor location. Both targets (‘X’ and ‘N’) appeared in each possible location 20 times, in each load condition. The ordering of the blocks was counterbalanced using an ABBA/BAAB format, evenly split across participants. The datasets generated and/or analysed are available from the corresponding author on reasonable request.

### Procedure

After obtaining informed consent, participants were seated at the computer to complete the example and practice trials. Participants then completed the four blocks of experimental trials. Each trial began with a fixation cross, which was presented for 500 ms, after which time the visual search array appeared for 100 ms. On distractor trials, distractors appeared alongside the visual search array, but remained visible until a response was made (to a maximum of 2000 ms). Participants were encouraged to respond as quickly and as accurately as possible. This procedure was identical to Forster and Lavie^2^.

## Results

### Distractor Presence

Accuracy data were initially examined to determine whether all participants performed above chance. Participants who performed below chance (Fisher’s binomial test = 54.6% for the current experiment) were excluded from analyses (*n* = 6). A 2 (perceptual load: low, high) ×2 (distractor presence: absent, present) within-participants analysis of variance (ANOVA) was conducted to determine the influence of distractors and perceptual load on accuracy. As anticipated there was a main effect of perceptual load, *F*(1,29) = 80.276, *p* < 0.001, η^2^ = 0.735. Accuracy rates were significantly higher in the low load condition (*M* = 89.46%, *SD* = 5.76), compared to the high load condition (*M* = 72.99%, *SD* = 9.97).

Although the main effect of distractor presence did not reach significance, *F*(1,29) = 3.881, *p* = 0.058, η^2^ = 0.118, the interaction between perceptual load and distractor presence was significant, *F*(1,29) = 7.133, *p* = 0.012, η^2^ = 0.197. In the low load condition, participants did not display any accuracy differences based on distractor presence, *t*(29) = 0.060, *p* = 0.953, *d* = 0.013 (distractor absent: *M* = 89.43%, *SD* = 5.35; distractor present: *M* = 89.47%, *SD* = 6.02). By contrast, participants showed slightly better performance when distractors were absent (*M* = 74.80%, *SD* = 10.35) relative to when they were present (*M* = 72.53%, *SD* = 10.09) in the high load condition, *t*(29) = 2.705, *p* = 0.011, *d* = 0.495. See Table 1 for accuracy rates.

**Table 1:** Means and Standard Deviations for Accuracy (% correct) and Reaction Time (ms).

Perceptual Load	Distractor	Accuracy (*SD*)	RT (*SD*)
Low	Lower	88.10 (*6*.*54*)	442.37 (*90*.*54*)
	Upper	89.60 (*6*.*86*)	439.58 (*88*.*96*)
	Left	89.93 (*7*.*64*)	425.97 (*87*.*41*)
	Right	90.23 (*7*.*04*)	427.90 (*87*.*22*)
High	Lower	71.53 (*10*.*55*)	583.47 (*199*.*88*)
	Upper	72.83 (*11*.*20*)	578.83 (*190*.*51*)
	Left	72.10 (*11*.*43*)	575.23 (*187*.*17*)
	Right	73.67 (*12*.*41*)	594.04 (*180*.*03*)

Next, we ran a 2 (perceptual load: low, high) ×2 (distractor presence: absent, present) within-participants ANOVA to determine the influence of distractors and perceptual load on reaction times. This analysis also determined whether we were able to replicate Forster and Lavie^2^. Incorrect responses were excluded from the reaction time analysis. There was a strong effect of perceptual load, *F*(1,29) = 134.299, *p* < 0.001, η^2^ = 0.822, indicating reaction times were faster during low load trials compared to high load trials. The main effect of distractor presence was non-significant, *F*(1,29) = 0.905, *p* = 0.349, η^2^ = 0.030.

**Figure 2:**
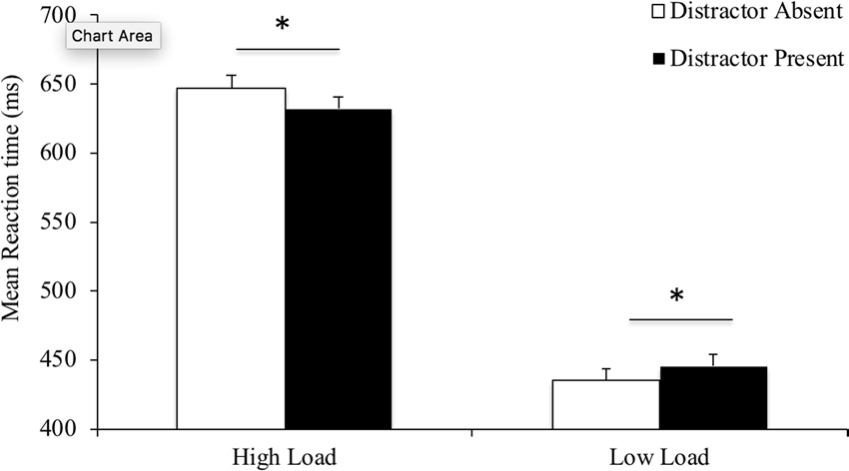
Interaction of perceptual load and distractor presence for present versus absent. Participants showed faster reaction times in the distractor absent trials compared to the distractor present trials during the low perceptual load condition. By contrast, participants were quicker in the distractor present trials during the high perceptual load condition. Error bars represent within-participant error bars, using standard errors of the mean.

Furthermore, there was an interaction of perceptual load and distractor presence, *F*(1,29) = 29.909, *p* < 0.001, η^2^ = 0.570 (see Fig. 2). In the low perceptual load condition, participants were faster on distractor absent trials as compared to distractor present trials, *t*(29) = 5.913, *p* < 0.001, *d* = 1.119. In contrast, for the high load condition, reaction times were quicker on distractor present trials than distractor absent trials, *t*(29) = 3.371, *p* = 0.002, *d* = 0.621. As participants showed a decrease in accuracy, as well as a decrease in reaction time for distractor present trials in the high load condition, the presence of a speed-accuracy trade-off is plausible. Given our primary question was: *does the location of the distractor differentially influence the size of the reduction in distractibility?* we do not want to over-interpret the interaction we observed here. Although our findings are consistent with Forster and Lavie^2^, they did not report a significant interaction. As such, we are cautious in interpreting the interaction we have observed.

Next, we explored the effect of distractor location separately for horizontal and vertical distractors. This analysis was chosen in lieu of running all possible contrasts, which could have returned statistically significant results that were of no theoretical interest.

### Vertical Distractors

To analyse accuracy for vertical distractors, we conducted a 2 (perceptual load: low, high) ×2 (distractor location: upper, lower) within-participants ANOVA. As expected, performance was superior under low load (*M* = 88.85%, *SD* = 6.16) compared to high load (*M* = 72.18%, *SD* = 10.02), *F*(1,29) = 0.70.208, *p* < 0.001, η^2^ = 0.708. Although perceptual load and distractor location did not interact, *F*(1,29) = 0.011, *p* = 0.917, η^2^ = 0.000, the main effect of distractor location was marginally significant, *F*(1,29) = 4.162, *p* = 0.051, η^2^ = 0.126. Given this effect did not reach statistical significance, and the mean difference in accuracy was slight this finding must not be over-interpreted; accuracy was slightly higher in the presence of upper space distractors (*M* = 81.22%, *SD* = 6.88) compared to lower space distractors (*M* = 79.82%, *SD* = 6.55).

**Figure 3:**
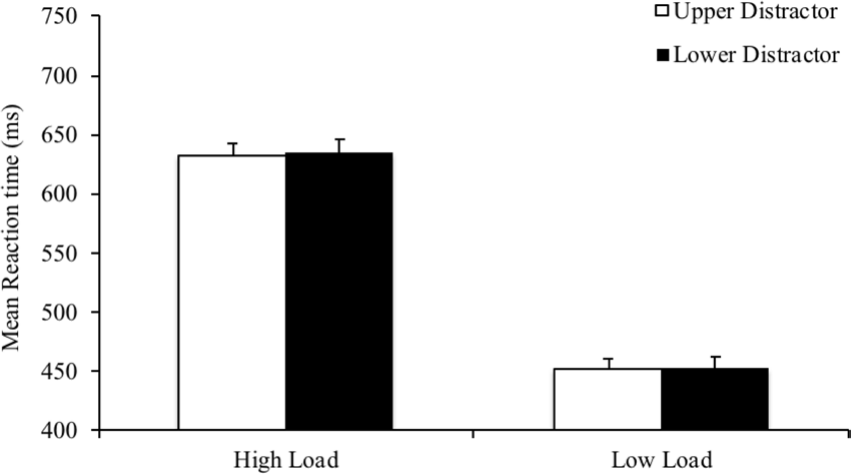
Interaction of perceptual load and distractor location, for vertically positioned distractors. There were no reaction times differences for upper as compared to lower distractors. Error bars represent within-participant error bars, using standard errors of the mean.

For vertical distractors, we expected upper space distractors to attract more attention and to decrease reaction times in the low load condition; however, in the high load condition, upper distractors were expected to lead to quicker reaction times. To investigate this effect, we conducted a 2 (perceptual load: high, low) ×2 (distractor location: upper, lower) within-participants ANOVA. There was once again a strong effect of perceptual load, *F*(1,29) = 94.953, *p* < 0.001, η^2^ = 0.766, which demonstrated that reaction times were significantly faster for low perceptual load trials (*M* = 452.32 ms, *SD* = 43.35), as compared to high perceptual load trials (*M* = 633.04 ms, *SD* = 115.79). The main effect of distractor location, *F*(1,29) = 0.100, *p* = 0.754, η^2^ = 0.003, was not significant, demonstrating there were no differences in reaction times between upper and lower distractors (see Table 1). Further, the interaction was non-significant, *F*(1,29) = 0.008, *p* = 0.929, η^2^ < 0.001 (see Fig. 3).

### Horizontal Distractors

For horizontal distractors, we first conducted a 2 (perceptual load: low, high) ×2 (distractor location: upper, lower) within-participants ANOVA for accuracy scores. Distractor location did not influence accuracy, *F*(1,29) = 0.814, *p* = 0.374, η^2^ = 0.027, and the interaction failed to reach significance, *F*(1,29) = 0.357, *p* = 0.555, η^2^ = 0.012. As above, there was a strong main effect of perceptual load, *F*(1,29) = 71.035, *p* < 0.001, η^2^ = 0.710. Once again, participants demonstrated superior performance under low load (*M* = 90.08%, *SD* = 6.66) compared to high load (*M* = 72.88%, *SD* = 10.23).

In relation to reaction time data, we expected a similar interaction between perceptual load and distractor location, such that left visual field distractors were expected to attract more attention than distractors to the right visual field. As a result, reaction times were expected decrease in the presence of left distractors in the low load condition but quicken in the high load condition. To investigate this effect, we conducted a second 2 (perceptual load: high, low) ×2 (distractor location: left, right) ANOVA.

**Figure 4:**
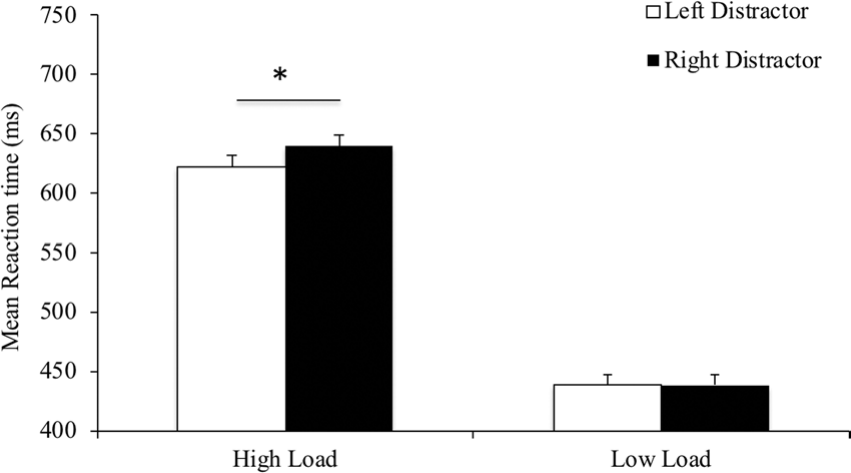
Interaction of perceptual load and distractor location, for horizontally positioned distractors. Reaction times were faster for left side distractors, as compared to right side distractors in the high load condition. By contrast, horizontally presented distractors did not differentially influence reaction times in the low load condition. Error bars represent within-participant error bars, using standard errors of the mean.

The main effect of perceptual load was significant, *F*(1,29) = 134.079, *p* < 0.001, η^2^ = 0.822, showing that participants were once again faster on the low perceptual load trials as compared to the high load trials. The main effect of distractor location failed to reach significance, *F*(1,29) = 3.594, *p* = 0.068, η^2^ = 0.110. Importantly, the interaction of perceptual load and distractor location was significant, *F*(1,29) = 4.601, *p* = 0.040, η^2^ = 0.137 (see Fig. 4). Reaction times were significantly faster for left, as compared to right, distractors in the high load condition, *t*(29) = 2.134, *p* = 0.041, *d* = 0.395 (see Table 1). This contrast was not significant in the low load condition, *t*(29) = 0.147, *p* = 0.884, *d* = 0.027. Importantly, we did not observe any differences in accuracy across these two conditions. As such, we can conclude that this difference is not the result of a speed-accuracy trade-off.

## Discussion

Using the irrelevant-distractor paradigm^2^, we manipulated distractor location to determine whether visual field differences in attention influence distractibility during visual search. As anticipated, our accuracy data illustrate that participants found the high perceptual load task to be significantly more difficult than the low load task. We replicated the findings of Forster and Lavie^2^, showing that participants were quicker to respond on low perceptual load trials, as compared to trials with a high load. While this finding is not surprising, it illustrates that the load manipulation was effective and participants performed the task as anticipated. When the visual search task was easier, participants were quicker to identify the target and respond.

More importantly, and consistent with Forster and Lavie^2^, distractor presence interacted with perceptual load. As described above, participants were quicker to identify targets when distractors were absent than when they were present in the low load condition. This finding is consistent with Forster and Lavie and illustrates that participants are more likely to be distracted by peripheral stimuli during tasks that engage fewer attentional resources. As the primary task in the low load condition was easier to complete, participants did not require all available attentional resources, which left additional capacity to process the irrelevant distractor figures.

In contrast, in the high load condition, reaction times were quicker in the presence, compared to the absence, of distractors. This finding supports the suggestion that when the primary task is more difficult and engages more resources, chances of distractibility are decreased. As there are fewer attentional resources available to process distractors, the spatial attentional window, or the “attentional spotlight”, narrows to focus on the primary task^39–41^. This narrowed focus is necessary for successful task completion and reflects efficient attentional engagement.

Expanding upon the findings of Forster and Lavie^2^, we tested whether the location of the distractors differentially impacted reaction times. In the low load condition, there were no location-based differences in performance. Participants had similar response times to left and right distractors, as well as upper and lower distractors. As there are known asymmetries in visuospatial attention, wherein individuals devote more attention to the left^15–18^, and upper^31,35^ visual fields, we believed distractors in these locations would attract more attention and consequently hinder performance to a greater extent. We failed to observe any such effect, which suggests participants were able to perform the primary task with ease and therefore had plenty of additional attentional resources to process the irrelevant distractors, regardless of location. Indeed, accuracy in the low load condition was at 85.80%, suggesting that the task was sufficiently easy for participants. Known asymmetries in visuospatial attention do not appear to influence distractibility for tasks that are low in perceptual load.

In contrast to the findings for low load, distractor location had a significant impact on distractibility in the high perceptual load condition. Indeed, distractor location only influenced performance when attentional resources were limited by the primary task. Although comparisons of distractibility for vertical distractors showed that upper and lower visual field distractors elicited similar performance advantages, horizontal distractor location differentially improved performance. Left visual field distractors led to a significantly greater performance advantage than did right visual field distractors.

Although we cannot determine the precise mechanism that reduces distraction during high perceptual load tasks, it is possible that the distractor operates as an alerting mechanism. This suggestion could also explain why left side distractors lead to the greatest decrease in reaction times. If participants exert a typical left-to-right scanning strategy, the occurrence of the left side distractor could increase alertness and cause participants to direct their attention toward the centre of the screen (i.e., the visual array) more quickly. Reductions in the attentional blink have been attributed to a centre-surround attentional mechanism^42–44^, wherein distractors that are similar to, but different from, the target reduce observed deficits. However, distractors that are sufficiently different from the target do not trigger this inhibitory mechanism and therefore do not lead a reduction in the attentional blink^42^, which suggests such a mechanism is unlikely to account for our findings. Instead, increased arousal or alertness as a result of attentional capture by the distractor appears more likely.

The neural mechanisms that underlie vertical and horizontal asymmetries are distinct, which could explain why left/right differences are observed, but upper/lower differences are not. This finding is in keeping with our hypothesis that left relative to right visual field distractors would capture attention and lead to a greater reduction in reaction time. Leftward biases are driven by right hemisphere activation during visuospatial attention tasks^45,46^, whereas upper and lower visual field differences are driven by relative activation within the ventral and dorsal visual streams^29,31^. As such, it would appear that the horizontal visual field is processed asymmetrically, whereas the vertical visual field is not. Right hemisphere activation during visual attention could explain the observed left side advantage, with our findings suggesting that relative activation within the dorsal and ventral visual stream does not provide an additional advantage in attentional control.

It is important to note that the number of distractor trials was necessarily low for each visual field location. We maintained an 80:20 ratio such that distractors were absent 80 per cent of the time and present 20 per cent of the time. In order to avoid participant fatigue, this ratio meant that the number of trials in each condition was low, which could have increase variability and resulted in additional noise in our data. We provide two interesting suggestions for future research that will further clarify the influence of visual field location on distractibility. Firstly, it would be interesting to vary the 80:20 ratio to determine whether the frequency of the distractors influences distractibility. Secondly, performing separate experiments to investigate the role of horizontal and vertical distractors would also allow for the number of distractor trials to be increased.

Leftward attentional asymmetries have been reliably observed for nearly 40 years^15,47^. Recent evidence^32–34^ suggests the strength of leftward biases is influenced by visual field differences in distractibility, such that upper visual field distractors increase the direction of attention to the left. Following from this work, we have demonstrated a significant performance improvement in the presence of irrelevant left side distractors, relative to equivalent distractors presented on the right, specifically when the task is highly demanding. This intriguing finding suggests that as the “attentional spotlight”^39–41^ narrows in the presence of distractors, it does so in an asymmetrical way.

Focused attention has been described as a spotlight, which narrows or expands as a function of perceptual load. The focus of the attentional window becomes sharper when tasks are more difficult and require more attentional resources, whereas attention is focused more broadly during easier tasks that do not require the same degree of attention^41,48–50^. One possible explanation for our data is we are able to engage a ‘catch and release’ style capture of attention more efficiently for left side distractors. As executive control is superior within the left visual field, we suggest that irrelevant distractors on the left initially capture attention more quickly than equivalent distractors on the right. Individuals are also able to disengage their attention more efficiently within the left visual field, allowing for distracting information on this side to be better ignored during high perceptual load. Our data suggest that attention is not focused symmetrically around the exact centre of the screen but shows superior ‘catch and release’ on the left side than on the right. Consequently, left side stimuli are more efficiently ignored and reaction times are quickest in the presence of left distractors.

This suggestion is similar to an idea proposed by Theeuwes, Atchley, and Kramer^51^, who found participants were able to exert sufficient attentional control over irrelevant distractors when stimulus onset asynchrony was 200 ms. Their findings show that attentional capture was not inhibited, but instead attention was successfully captured and then controlled by the participants. Interestingly, the top-down control that was observed at 200 ms, was not observe for longer stimulus onset asynchronies (i.e., 400 ms), which suggests the inhibition that occurs as a result of top-down control is likely transient and short lived^51^. A similar mechanism, such as the catch and release attentional capture we have proposed, could explain our findings—with the main difference relating to the presentation time of the stimuli. Given our distractor and visual search array were presented simultaneously for 100 ms, our data suggest that it is possible to engage a certain level of attentional control when stimuli are presented briefly.

Interestingly, there is evidence that our attention can be split across two spatial locations, in situations where two stimuli must be processed in close succession^49^. As such, an alternative explanation for the current findings would be a split in attention, allowing cartoon distractors as well as the visual search array, to be processed. In this instance, both the array and the distractor would be processed; however, if disengagement of attention within the left visual field is superior, it would allow participants to ignore these distractors more easily. Such an advantage would be consistent with known visuospatial asymmetries, wherein attentional control is superior in the left visual field as a result of right hemisphere activation^16–18,45,46^. Further, neuroimaging has shown extensive right hemisphere activation during disengagement^52–56^, which suggests this process could be facilitated within the left visual field. In keeping with this suggestion, a sub-division of the attentional spotlight would occur for all distractors, explaining why processing was more efficient in the presence of distractors, regardless of location, during high perceptual load; however, disengagement in all other locations would be slightly slower, leading to a smaller reduction in reaction times as compared to the left side. Future research, which makes use of electrophysiological measures and eye tracking, is needed to confirm this suggestion.

## Conclusion

We found that distractor presence interacted with perceptual load, which replicates Forster and Lavie^2^. In the low load condition, the task was easier, which allowed participants to respond more quickly when distractors were absent than when they were present. In contrast, when the primary task was more difficult in the high load condition, more attentional resources were engaged, and the chances of distractibility were decreased. Extending upon this result, we manipulated distractor location to determine whether left as compared to right, and upper as compared to lower, visual field distractors differentially influence visual search performance. During low perceptual load, the focus of attention was sufficiently large to incorporate both the visual search array and the distractor figure, which allowed all distractors to draw attentional resources away from the search array and slow reaction times. Therefore, during low perceptual load divided attention is not required, leading distractors and the visual search array to share attentional resources, which causes increased distraction.

In contrast, attentional resources are limited during high perceptual load, which decreases chances of distractor processing. We failed to observe a performance difference when comparing upper and lower visual field distractors during high perceptual load; however, left distractors improved performance significantly more than equivalent right-side distractors. A split in the attentional spotlight could explain why distractors in all other locations also decrease reaction times during conditions of high load, as compared to trials with no distractors. Alternatively, we suggest that executive control is better in the left visual field, allowing left distractors to be ‘caught, released’ and consequently ignored, more efficiently, leading a greater performance benefit when distracting information is on the left side during highly demanding tasks.
